# The Utility of Repetitive Cell-Free DNA in Cancer Liquid Biopsies

**DOI:** 10.3390/diagnostics12061363

**Published:** 2022-06-01

**Authors:** Ugur Gezer, Abel J. Bronkhorst, Stefan Holdenrieder

**Affiliations:** 1 Department of Basic Oncology, Institute of Oncology, Istanbul University, 34093 Istanbul, Turkey; 2 Munich Biomarker Research Center, Institute of Laboratory Medicine, German Heart Center, Technical University Munich, 80636 Munich, Germany; bronkhorst@dhm.mhn.de (A.J.B.); holdenrieder@dhm.mhn.de (S.H.)

**Keywords:** cancer, liquid biopsy, repeat elements, ALU, LINE-1, satellite DNA, cell-free DNA

## Abstract

Liquid biopsy is a broad term that refers to the testing of body fluids for biomarkers that correlate with a pathological condition. While a variety of body-fluid components (e.g., circulating tumor cells, extracellular vesicles, RNA, proteins, and metabolites) are studied as potential liquid biopsy biomarkers, cell-free DNA (cfDNA) has attracted the most attention in recent years. The total cfDNA population in a typical biospecimen represents an immensely rich source of biological and pathological information and has demonstrated significant potential as a versatile biomarker in oncology, non-invasive prenatal testing, and transplant monitoring. As a significant portion of cfDNA is composed of repeat DNA sequences and some families (e.g., pericentric satellites) were recently shown to be overrepresented in cfDNA populations vs their genomic abundance, it holds great potential for developing liquid biopsy-based biomarkers for the early detection and management of patients with cancer. By outlining research that employed cell-free repeat DNA sequences, in particular the ALU and LINE-1 elements, we highlight the clinical potential of the repeat-element content of cfDNA as an underappreciated marker in the cancer liquid biopsy repertoire.

## 1. Introduction

The term circulating nucleic acids (cirNAs) refers to segments of genomic DNA, mRNA, and noncoding RNAs in the cell-free fraction of blood (i.e., serum or plasma), and has been receiving increasing attention as a source of biomarkers, especially in oncology [1,2,3]. Although cirNAs also encompass various species of RNAs, it usually refers to double-stranded DNA, called cell-free DNA (cfDNA). Although the history of cfDNA dates back to the 1940s, much progress in the understanding of its origin and composition and the extensive potential as a minimally invasive source of diverse pathologic conditions has been made in the last two decades [4]. In 1989, the Stroun group demonstrated that a fraction of the plasma cfDNA in patients with cancer was derived from cancer cells, which is based on the presence of cancer cell DNA with decreased strand stability in plasma samples of patients with cancer [5]. Shortly thereafter, TP53 mutations were detected in urine from patients with invasive bladder cancer [6]. The subsequent surge of studies confirmed that cancer cells released detectable concentrations of cfDNA into circulation, and more importantly, that a proportion of cfDNA fragments harbored unique genetic and epigenetic alterations of the tumor cells from which they derived [4]. This unequivocal proof that a fraction of cfDNA was derived from cancer cells prompted the research efforts around cfDNA, which is now widely recognized as a promising biomarker in cancer screening and monitoring of the efficacy of anticancer therapeutic strategies [7]. Currently, established clinical use of cfDNA liquid biopsy tests include: (i) FoundationOne Liquid CDx, (ii) COBAS EGFR mutation test V2, (iii) Therascreen PIK3CA RGQ PCR, (iv) Guardant360 CDx, and (v) Epi proColon, for SEPT9 methylation detection in plasma [8,9]. Here, it is noteworthy that the fundamental importance of understanding, studying, and analyzing germline DNA before performing liquid biopsy assays is increasingly recognized, as this will enable enhanced differentiation between constitutional genetic alterations and somatic alterations (e.g., due to cancer).

In contrast to the substantial clinical interest in cfDNA, its characterization has initially received little attention. Thus, a lack of understanding of its composition impaired the elucidation of cfDNA biology and its potential clinical use [10]. In the 2000s, conventional cloning and DNA-sequencing techniques were employed to characterize cfDNA and its composition [11,12]. However, such labor-intensive methods can generate limited sequence information due to their inability to cover all sequences of cfDNA [13].

The introduction of molecular barcodes has enhanced the sensitivity of sequencing methods. This and further advancements in sequencing technologies along with the progression of bioinformatics facilitated the accurate characterization of cfDNA composition [14,15]. As a consequence, sequencing efforts of cfDNA have increased considerably in recent years and several studies have not only described clinically relevant genetic mutations in cancer patients at various stages of the disease [16,17,18], but have demonstrated correlations between the pathology and progression of cancer and various epigenetic features of cfDNA molecules, including various fragmentation features (i.e., size signatures, preferential cleavage sites, jagged ends, unique fragment end-point motifs, orientation-aware fragmentation patterns, nucleosome spacing and density, and topological features) (reviewed in [19]), methylation patterns, and post translational histone modifications (reviewed in [20,21]).

**Figure 1:**
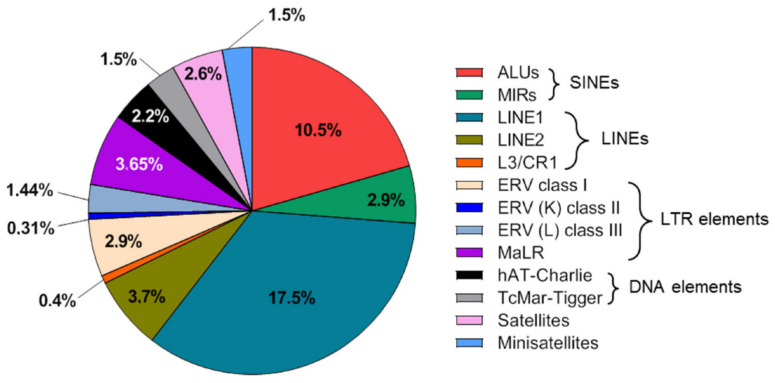
Distribution of the major repetitive element families in the human genome. The repetitive element landscape is vulnerable to numerous cancer-specific modifications. The abundance of repetitive DNA in the human genome and its widespread occurrence across all chromosomes, is mirrored in the cfDNA content of clinical biospecimens, which makes the characterization of repetitive cfDNA an attractive liquid biopsy tool for interrogating various cancer indications.

**Figure 2:**
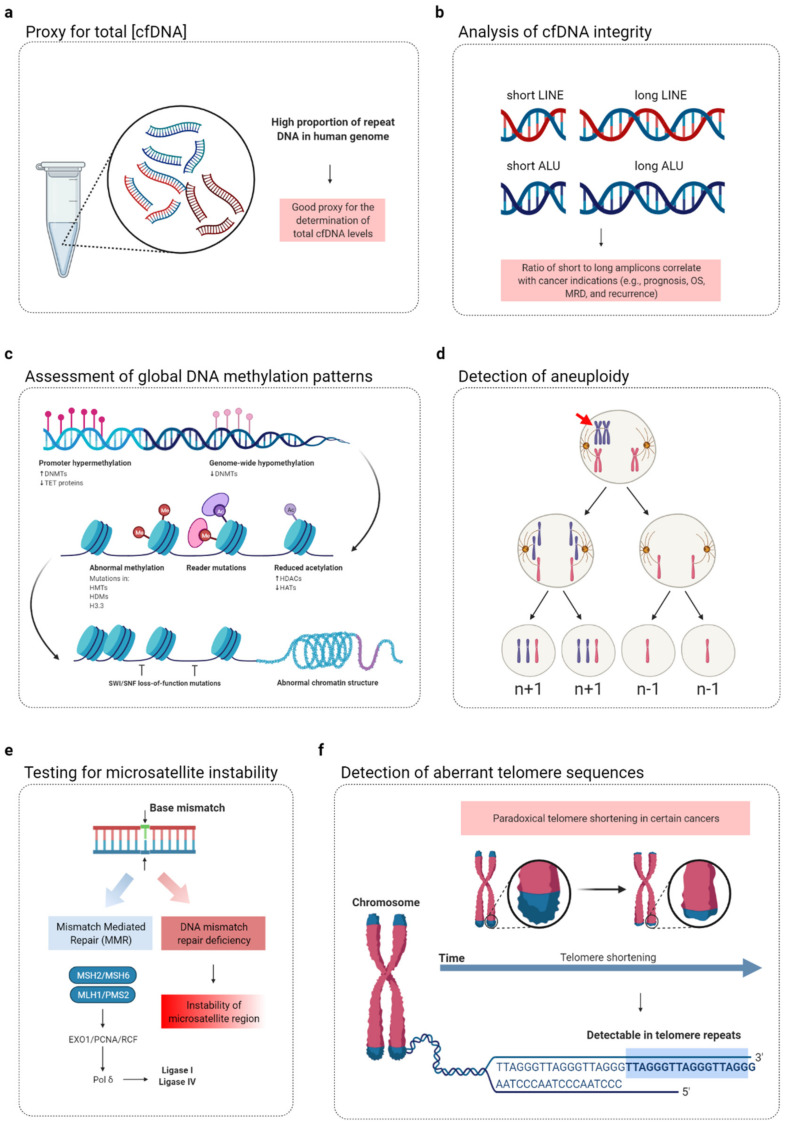
Potential applications of cfDNA repeat element analysis in cancer liquid biopsies. The repetitive element content of cfDNA can be used to characterize various features of cancer, including: (**a**) increased total cfDNA levels; (**b**) changes in cfDNA fragment size; (**c**) global DNA methylation changes; (**d**) aneuploidy; (**e**) microsatellite instability; and (**f**) aberrant telomeric sequences. This represents an intriguing modality for the use of cfDNA to manage cancer patients and may complement many of the current and newly emerging cfDNA profiling strategies.

Owing to improvements in profiling the composition of cfDNA, several studies have recently demonstrated the significant potential of the repeat element DNA portion of cfDNA in liquid biopsies of cancer. In this review, after a brief description of the repeat content of the human genome and cfDNA (Figure 1), we highlight the various potential applications of cfDNA repeat analysis as a surrogate marker for various indications in cancer (Table 1 and Figure 2) and summarize research in which repeat DNA sequences in liquid biopsy were used in patients with cancer (Table 2).

## 2. Repeat DNA Content of the Human Genome and Cell-Free DNA

Repeat DNA is usually defined as DNA present in multiple copies in the genome and a common feature of eukaryote genomes. More than 50% of the human genome is composed of repeat DNA [22], with some estimates as high as two-thirds of the genome [23]. With respect to their genomic distribution, repeat DNA elements can be divided into two groups: tandem repeats and interspersed repeats. Tandem repeats, which account for up to 6% of the human genome, are repetitions of the same sequence motifs aligned in a head-to-tail fashion and cover a significant fraction of heterochromatin and centromeric regions. Microsatellites, minisatellites, centromeric/pericentric satellites, and telomeric/subtelomeric repeats are members of the tandem repeat group. Microsatellites are tandem repetitions of short (1–9 bp) units and are of clinical relevance because their instability (i.e., hypermutability) as a consequence of the loss of mismatch DNA repair is a feature of some human cancers such as colorectal cancer [24]. Minisatellites are a class of highly polymorphic GC-rich tandem repeats consisting of 10–100-bp units and include some of the most variable loci in the human genome, with mutation rates ranging from 0.5% to >20% [25]. Centromeric/pericentric satellite sequences, which account for approximately 3% of the human genome, are constituents of centromeric and pericentromeric heterochromatin and telomeres and have been implicated in chromosome organization and segregation, kinetochore formation, as well as heterochromatin regulation [26]. α-satellites, representing repetitions of 171 bp units assembled into higher-order structures, are highly abundant centromeric elements. Human satellite 2 (HSATII) is an approximately 26-bp tandem repeat and is found in small blocks on the pericentromeres of several human chromosomes [27]. α-satellites and HSATII have been documented to be highly expressed in tumor cells, which leads to their reverse transcription and stable reintegration into the human genome, expanding their genomic copy numbers [28]. Interspersed repeats are considered to be remnants of transposable elements (TEs) and constitute approximately 45% of the human genome [29]. Retrotransposable elements (RTEs) are primary components of TEs and can proliferate and insert themselves into new genomic regions. RTEs are classified into long terminal repeat (LTR) elements and non-LTR elements, which differentiate in the mechanism of retrotransposition and the possession of long terminal repeats [24]. The non-LTR elements are categorized as either long interspersed nuclear elements (LINEs) or short interspersed nuclear elements (SINEs) [30], which are predominantly represented by the LINE-1 and ALU families, respectively. The relative proportion of the major classes of repetitive elements in the human genome, as determined by masking of the human genome using RepeatMasker, is shown in Figure 1.

As repeat DNA makes up more than 50% of the human genome, it would be expected to also constitute the major part of cfDNA fragments. The first report describing the repeat content of cfDNA dates back to 2009 in which Beck et al. sequenced serum cfDNA of healthy individuals [31]. They found that most classes of sequences (e.g., genes and RNA and DNA coding sequences) did not differ between serum DNA and genomic DNA. Of the repeat elements, ALU sequences constituted a higher proportion of cfDNA with statistical significance, whereas LINE-1 and LINE-2 element sequences were found to be present in lower proportions [31]. In a similar article in which cfDNA from healthy individuals and patients with cancer was sequenced using parallel tagged sequencing on the 454 platform, the majority of repeat sequences in patients, as well as controls, were either LINE-1 or ALU repeats because they represent almost half of the total repeat count, where the repeat content of cfDNA was slightly higher in controls than in patients with cancer (46% vs. 42%). A substantial number of satellite sequences were also detected in both groups. All the repeat classes had a slightly higher representation in the control group than in the cancer group [32].

In an in-vitro study, Bronkhorst et al. [33,34] investigated the composition of cfDNA released into the growth medium by cultured osteosarcoma cells. The sequencing of cfDNA revealed that cfDNA consisted mainly of TEs, α-satellites, and minisatellites. Interestingly, a major portion of these repeat element sequences were found to derive from chromosomes 1 and 9, where the authors postulated that increased instability as a result of increased hypomethylation in centromeric and pericentromeric regions of chromosomes 1 and 9 could be the mechanism of selective release of such repeat families from cells into culture medium [34]. In a more recent study employing high-throughput sequencing of cfDNA, it was demonstrated that there were significant differences between the composition of cfDNA in serum/plasma and the corresponding DNA sequence composition of the human genome [35]. Compared with their genomic abundance, retrotransposable elements and pericentric satellite DNA were found to be particularly overrepresented in the cfDNA population, and telomeric satellites were underrepresented. The authors explained this overrepresentation of repeat families as a consequence of reverse transcription of retrotransposable elements and reintegration and secondary-structure formation during the replication of satellite DNA contributing to the composition of the cfDNA molecules in the mammalian bloodstream [35]. The differences that emerged between different studies with respect to repeat content of cfDNA may be attributed to many variables such as the use of different pre-analytical steps, experimental procedures, or sequencing platforms.

The study of cfDNA fragmentation patterns, also referred to as ‘fragmentomics’, is a rapidly evolving area of research [19]. Fragmentation analysis of sequenced cfDNA is useful for shedding light on emerging markers, such as fragment sizes, preferred ends, end-motifs, single-stranded jagged ends, and nucleosomal footprints. Given the central role of chromatin structure (and by extension the repeat element content of genomic regions) in dictating DNA digestion/degradation activities, incorporating the analysis of repetitive cfDNA, which is predominant in heterochromatin that makes up the majority of the human genome, into the fragmentomics toolbox could provide further insights into the role of repetitive DNA in different physiological states and cancer. Such integrated fragmentomics analyses will also enable a better understanding of the differential abundance and profiles of repetitive cfDNA in various cancer types.

## 3. Application of Cell-Free Repetitive DNA in Liquid Biopsies of Cancer

### 3.1. Quantification of Total Cell-Free DNA Using Repeat Elements

Extraction of cfDNA from plasma and other body fluids is one of the first and most important steps in liquid biopsy tests. However, there is currently no gold standard, and several issues challenge the optimal isolation of cfDNA. Two drawbacks in particular may significantly influence the analysis of repetitive elements in cfDNA. First, the concentration of cfDNA in healthy subjects and cancer patients (~30 ng/mL, depending on tumor type and burden) is relatively low [36], which negatively impacts the sensitivity and precision of downstream analyses. This is especially true for some manual extraction kits and automated methods that do not deliver a high yield of cfDNA [37,38]. Second, increasing evidence shows that the cfDNA population in a typical biospecimen is highly diverse with respect to fragment size. While a major portion of cfDNA molecules are present as mono-nucleosomal fragments [31], sub-nucleosomal fragments, oligo-nucleosomes, circular DNA, mitochondrial DNA, and high molecular weight cfDNA is most often co-present. As the repeat content of differently sized cfDNA populations has not yet been elucidated, it is highly likely that the repeat content of the cfDNA measured in a sample is influenced significantly by the selected extraction kit, as different methods have demonstrated significant bias toward the capture or exclusion of specific fragment sizes [39]. Therefore, robust methods of sample preparation in liquid biopsies are required to reduce sample preparation errors and aid the standardization of cfDNA extraction. To our knowledge, there are no specialized techniques or kits to enrich cell-free repetitive DNA, while the typical commercially available cfDNA extraction protocols of columns or bead-based kits have been followed by research laboratories for handling repetitive cfDNA (Table 1 and Table 2).

Quantitative polymerase chain reaction (qPCR) has been employed in numerous studies to quantify miniscule amounts of cfDNA in bodily fluids. However, the amplification of repetitive elements using PCR may often be challenging, as the abundance and sequence features of target repetitive DNA have to be considered for designing primers. Moreover, possible polymorphic sites on the target sequence may result in false identification and amplicons of multiple lengths are expected, thereby making it difficult to make concrete interpretations of the results [40].

**Table 1:** An overview on applications of repetitive DNA elements in liquid biopsy.

Repeat Name	Repeat Type	Liquid Biopsy Application in Patients with Cancer	References
LINE1	Interspersed	Quantification of total cfDNA	[41,42,43,44]
		Assessment of cfDNA integrity	[45]
		Detection of aneuploidy by amplicon sequencing	[46,47,48]
		Assessment of cfDNA global methylation status	[49,50,51,52,53,54,55,56,57,58,59]
ALU	Interspersed	Quantification of total cfDNA	[49,60,61,62,63,64,65,66,67,68]
		Assessment of cfDNA integrity	[44,62,63,66,67,69,70,71,72,73,74,75,76,77,78,79]
		Detection of aneuploidy by amplicon sequencing	[48]
Human satellite 2	Tandem	Quantification of total cfDNA	[80]
Microsatellites	Tandem	Assessment of microsatellite instability via cfDNA	[81,82,83,84,85,86,87]
Telomeric repeats	Tandem	Quantification of telomeric cfDNA	[88,89]
		Assessment of telomere length in cf DNA	[90,91]

LINE-1 and ALU have been the most used repeat elements for cfDNA quantitation. Given that generally higher cfDNA concentrations are found in patients with cancer than in controls using the quantification of non-repeat DNA, similarly, several studies employing LINE-1 or ALU as the target sequence found higher levels of cfDNA in serum or plasma of patients with cancer including breast [41,49,60], lung [42,61,62], prostate [63,64,65], colorectal [66,67], and gastric cancers [68], and neuroblastoma [43]. In many cases, elevated cfDNA concentration was associated with tumor burden/advanced tumor stage [41,42,43,62,67]. Many studies reported the prognostic value of repeat cfDNA in different cancer types including breast [45,92], lung [62], prostate [65], and colorectal cancers [67], where generally increased cfDNA levels were associated with poor prognosis.

In addition to diagnostic and prognostic value, cell-free repeat element DNA was found to be useful in the monitoring of patients with cancer. ALU cfDNA levels were significantly decreased after the third cycle of chemotherapy compared with baseline levels in patients with breast cancer [49]. In gastric cancer, post-surgical high concentrations of LINE-1 were found to be indicative of minimal residual disease and recurrence risk [50]. In prostate cancer, LINE-1-based cfDNA measurement was found to be useful in determining dynamic changes in response to cytotoxic chemotherapy [93]. Repeat DNA was also used in the quantitation of cfDNA using the lab-on-a-chip approach. Following magnetic probe labeling, ALU sequences were employed in the detection of cfDNA using an array of magnetoresistive (MR) sensors integrated into a portable biochip platform in patients with cancer [94].

Even if pericentric satellites comprise less than 1% of the human genome, they are the most overrepresented repeat (2230%) in the cfDNA population in healthy individuals when compared with their genomic abundance [35]. In addition to this amazing feature of HSATII-specific cfDNA fragments, HSATII has been shown to expand in copy number in tumor cells via RNA-derived DNA intermediates [28]. These two conditions, i.e., overrepresentation in cfDNA in physiologic conditions and the expansion in copy number in tumor cells makes this repeat element an ideal marker for liquid biopsy of patients with cancer. In line with this expectation, in a recent exploratory study [80], we amplified HSATII repeats from chr1, 10, and 16, and found higher plasma levels of HSATII over LINE-1 in patients with cancer. Intriguingly, a chromosome 10-associated HSATII repeat sequence tended to give the best results. Another advantage of such overrepresented repeat sequences in cfDNA would be its direct amplification from serum/plasma without a need for DNA extraction from serum or plasma. In our study, we were able to amplify HSATII from diluted plasma and the quantification of the chromosome 10-associated HSATII repeat sequence from diluted plasma in a small cohort including breast, lung, colon, and gastric cancers, sarcoma, and lymphoma, which resulted in an area under the curve value of up to 94% [80]. As various preanalytical steps unambiguously affect the outcomes of cfDNA measurements, and DNA extraction kits from different manufacturers result in different cfDNA concentrations [95,96,97], employing diluted plasma as a template instead of extracted DNA may reduce variations and any bias in cfDNA measurements in future studies.

**Table 2:** Studies that utilized cell-free repetitive DNA in clinical use of cancer patients.

Clinical Use	Repeat Type	References
Evaluation of diagnostic potential of cedant	LINE1	[41,42,43,44,46,47,48,49,51,52,54,55]
	ALU	[44,49,61,62,63,64,65,66,67,68,69,70,71,72,74,75,76,77,79,98,99]
	Human satellite 2	[80]
	Microsatellites	[81,82,85,86]
Prognostic significance of cfDNA	LINE1	[42,44,45,50,53,54,56,57,59]
	ALU	[62,66,67,71,72,73]
	Microsatellites	[83]
Predicting the response to neoadjuvant chemotherapy	ALU	[100,101,102]
Monitoring of cancer patients	LINE1	[42,50,53,55,56,57]
	ALU	[60,64,68,79]
	Microsatellites	[85,87]

### 3.2. Analysis of Cell-Free DNA Integrity Using Repeat DNA

Owing to their high abundance in the population of cfDNA fragments compared with non-repeat DNA, another application of repeat element DNA has been the assessment of the integrity of serum/plasma DNA. Over the last two decades, the analysis of cfDNA integrity has emerged as a subject of efforts for developing cfDNA-based biomarkers in cancer diagnosis and prognosis [103], because it is an independent factor from the genetic or epigenetic status of cfDNA and is theoretically representative of all tumors [104]. The mechanism of cfDNA released into the bloodstream is the primary determinant of fragmentation signatures of serum/plasma DNA. Fundamentally, non-neoplastic cells that undergo apoptotic cell death, shed DNA fragments of nearly 180–200 bp in size as a result of enzymatic cleavage of nucleosome units, whereas tumor cells undergo many different death processes, including necrosis and autophagy, and can release longer DNA fragments [105,106]. For years, cell death, primarily via apoptosis or necrosis, has been considered to be the only relevant mechanism of DNA released into the bloodstream [14], as justified by the non-random fragmentation pattern of circulating cfDNA [107], where this non-random fragmentation process was found to be associated with the positioning of nucleosomes [108,109]. However, besides cell death-associated DNA released into blood circulation, an active release of cfDNA from living cells has also been described [110].

cfDNA integrity is calculated as the ratio of the concentration of longer DNA fragments to shorter fragments in plasma or serum. ALU elements are the most used repeat DNA elements in the assessment of size analysis of cfDNA. Umetani et al. [69] were the first to employ ALU sequences for this purpose. They measured shorter fragments of 115 bp as representatives of those fragments derived from apoptotic normal cells and larger ones of 247 bp as representatives of cfDNA derived from necrosis/autophagy of cancer cells. The ALU247/ALU115 ratio was found to be higher in patients with breast cancer with high-grade disease compared with healthy controls and suitable-to-define lymph node metastasis [69]. Thereafter, numerous studies used ALU and/or LINE-1 elements to assess cfDNA integrity in breast [70,71,72] and other cancers including lung [62,73], colorectal [66,67,74], prostate [63,72], endometrial [75], bladder [76], ovarian [77], thyroid cancers [98], and hepatocellular carcinoma (HCC) [79]. Apart from the diagnostic value, many studies have described a prognostic role of cfDNA integrity using repeat DNA. Repeat cfDNA integrity was shown to be an independent prognostic marker for survival in primary and metastatic breast cancer [44,111]. In patients with non-small cell lung cancer, those with low ALU cfDNA integrity had better overall survival (OS) [62]. In prostate cancer, ALU cfDNA integrity increased with disease severity and higher staging [72].

In principle, increased release of longer fragments by tumor cells via apoptosis results in an increase of the cfDNA integrity in patients with cancer. However, the findings from studies across many cancer types are not consistent, and both increased and decreased DNA integrity has been described in patients with cancer compared with controls [45,72]. Even if numerous studies reached statistically significant differences in cfDNA integrity index between patients with cancer and control individuals, this approach has limited diagnostic/screening potential because DNA fragments derived from tumor cells are vastly diluted by normal DNA. Despite this limited diagnostic value, the assessment of cfDNA integrity potentially represents a useful tool for the monitoring of patients with cancer [112]. cfDNA integrity has been evaluated for several purposes in patients with cancer such as the assessment of recurrence risk and the response to cytotoxic therapy or immunotherapy. Patients with breast cancer with a lower cfDNA integrity of ALU/LINE-1 sequences were found to have a much higher risk of developing recurrence than those with a higher cfDNA integrity [45]. In colorectal cancer, the serum ALU DNA integrity index proved to be a promising candidate biomarker for prognostic prediction of patients who underwent chemotherapy and during short-term follow-up [99]. cfDNA size analysis using ALU elements has also been evaluated in the course of cytotoxic therapy both in the neoadjuvant or adjuvant setting in breast cancer [100,101], or rectal cancer [102]. In patients with breast cancer with locally confined disease who underwent neoadjuvant chemotherapy, the kinetics of plasma DNA (ALU 115) from cycle 1 to 6 were found to be associated with the response to neoadjuvant chemotherapy [101]. In rectal cancer, longer ALU fragments and the cfDNA integrity index were found to be promising markers to predict tumor response to neoadjuvant chemotherapy [102]. In patients with advanced non-small-cell lung cancer during treatment with personalized peptide vaccinations, cfDNA integrity of ALU element was decreased after the first vaccine cycle, and the patients with high prevaccination cfDNA integrity survived longer than those with low prevaccination integrity (median survival time: 17.9 vs. 9.0 months) [113]. Similar results have been obtained in endometrial cancer where the cfDNA integrity was decreased after the first vaccine cycle, and the decreased cfDNA integrity levels were correlated with vaccine-induced immune responses [114]. A decrease in ALU cfDNA integrity was more frequent in IgG-positive or cytotoxic T cell response-positive patients, which suggested cfDNA integrity as a possible biomarker for cancer vaccine therapy. These findings suggest that the integrity assessment of circulating repeat sequences is a useful tool that may be integrated into personalized cancer management.

### 3.3. Detection of Aneuploidy in Patients with Cancer Using Cell-Free Repeat DNA

Aneuploidy is defined as an abnormal chromosome number and was the first genomic abnormality identified in human cancers [115]. Aneuploidy has been estimated to be present in >90% of cancers [116]. Karyotyping was the first technique for the detection of aneuploidy on the chromosome level. Subsequently, Sanger sequencing, microarrays, and most recently, massively parallel sequencing methods, have been used for the determination of copy number variations in cancers. Amplicon-based sequencing protocols were also used for aneuploidy detection [46] and achieved high coverage depth at relatively low cost and required relatively low amounts of template DNA. Thus, amplicon-based protocols are attractive alternatives to whole-genome sequencing [47,48].

In the first use of repeat elements in aneuploidy detection, a single primer was used to amplify 38,000 LINE sequences across the genome using the FAST-SeqS approach [46]. Using this approach, samples containing as little as 4% trisomy 21 DNA could be readily distinguished from euploid samples. In a refinement of the FAST-SeqS approach, Douville et al. [47] developed a method to evaluate the sequencing data obtained from LINE amplicons. This method, called within-sample aneuploidy detection (WALDO), employed supervised machine learning to detect gains and losses in multiple chromosomes in cancers, and aneuploidy was detected in 95% of 1677 tumors and 22% of 1522 liquid biopsies [47]. In the next step, the same group [48] developed a PCR-based assay called the repeat element aneuploidy sequencing system (RealSeqS) to detect aneuploidy in cfDNA in samples containing as little as 3 pg of DNA. In this sophisticated assay design, using a single primer pair based on short repeats in LINEs/ALUs, they amplified nearly 350,000 amplicons distributed throughout the genome, which enabled the detection of aneuploidy in 49% of samples from 883 nonmetastatic, clinically detected cancers of the colorectum, esophagus, liver, lung, ovary, pancreas, breast, and stomach [48].

### 3.4. Assessment of Global DNA Methylation Status of Cell-Free DNA Using LINE-1 Elements

Aberrant DNA methylation including regional hypermethylation and global hypomethylation is the most common molecular lesion of the cancer cell [117]. Global DNA hypomethylation, defined as decreased 5-methylcytosine content in genomic DNA, is a frequent epigenetic abnormality in cancer and a characteristic of repeat sequences that are highly methylated in normal cells, such as retrotransposons (ALU and LINE) and centromeric satellite DNA. DNA hypomethylation is associated with genomic instability promoting tumor progression because it can significantly elevate mutation rates and DNA recombination [118,119]. A meta-analysis of the results of 20 studies with a total of 5447 patients with cancer that employed LINE-1, ALU, or Sat-α repeat elements in the assessment of global hypomethylation found that global DNA hypomethylation was associated with a detrimental prognosis of patients with cancer [120].

Compared with hypermethylation of tumor suppressor genes, the assessment of hypomethylation of repeat elements in cfDNA has attracted less attention [121]. The methylation status of particularly LINE-1, which makes up 17% of the human genome, is considered to be an excellent indicator of the global DNA methylation status because retrotransposons constitute a significant portion of the human genome [22,122]. Due to the low amount of tumor DNA in circulation, a large volume of plasma is needed to ensure the sensitivity of the circulating tumor DNA-based assay. For instance, methylated SEPT9-based test Epi proColon requires 3.5 mL of plasma. By contrast, due to the high abundance of LINE-1 in the human genome, only 0.5 mL of plasma is sufficient to quantify LINE-1 methylation levels [51].

Several studies assessed the global methylation status of cfDNA via LINE-1, and LINE-1 hypomethylation has been described in many cancers such as breast [52], colorectal [51,53], HCC [54], esophageal adenocarcinoma [55], and oral cavity cancer [56]. In patients with malignant melanoma, an increase of LINE-1 hypomethylation was observed in the cfDNA from sera of patients with stage III and IV disease compared with healthy donors [57]. In a recent study [49], LINE-1 methylation status combined with ALU cfDNA integrity was found to be useful to discriminate patients with breast and lung cancer from healthy individuals. In the HER2-enriched subtype of breast cancer, an aggressive entity of breast cancer, hypomethylation of ALU and LINE-1 was shown to be a prominent molecular event [58]. Methylation of LINE-1 in cfDNA was also found to have prognostic value in some cancers. In colorectal cancer, patients with LINE-1 hypomethylation had significantly worse progression-free (median: 6.6 vs. 9.4 months; *p* = 0.02) and overall (median: 16.6 vs. 23.2 months; *p* = 0.01) survival following chemotherapy compared with patients with high methylation, and LINE-1 hypomethylation was an independent factor for poor prognosis. Additionally, LINE-1 hypomethylation was associated with a trend for non-response to chemotherapy with the FOLFOX regime [53]. In gastric cancer, pre-surgical low methylation levels of LINE-1 were found to be a negative prognostic factor, whereas post-surgical high concentrations of LINE-1 were indicative of minimal residual disease (MRD) and a high risk of recurrence [50]. In HCC, multivariate analyses showed that serum LINE-1 hypomethylation was a significant and independent prognostic factor of OS in patients with HCC [54]. Similarly, high serum LINE-1 hypomethylation was shown to correlate significantly with poor survival of patients with HCC [59].

### 3.5. Assessment of the Microsatellite Instability via Cell-Free DNA

Microsatellites, also known as simple sequence repeats, are defined as 10–60 bp regions that contain multiple repeats of 1–9 bp motifs in tandem [123]. They are widely spread throughout the genome and located both in coding and non-coding regions. Microsatellite instability (MSI) is defined as a hypermutable state occurring at microsatellites and is caused by defects in the mismatch repair (MMR) system. Impairment of the MMR system can render cells unable to regulate the lengths of their microsatellites during cell division and after multiple cycles of cell division, cells with an impaired MMR system will develop varying lengths in their microsatellite sequences [124].

MSI has been frequently observed in several cancer types, most commonly in colorectal, endometrial, and gastric adenocarcinomas [125]. A study from 2017 investigated the MSI in more than 11,000 tissue samples across 39 cancer types. Twelve cancer types were found with an MSI prevalence greater than 1%, mostly represented by endometrial (31.4%), gastric (19.1%), and colorectal adenocarcinomas (16.0%) [126]. The clinical significance of MSI has been well described in colorectal cancer; patients with MSI-H (MSI-high) tumors have been shown to have favorable prognosis compared with those with microsatellite stable tumors. In addition, it has a role as a biomarker for familial cancer risk assessment and cancer prognosis; MSI status was found to predict the response to immune checkpoint inhibitors such as the programmed cell death 1 (PD-1) inhibitor [127].

The detection of MSI in cfDNA goes back to 1996; two groups reported microsatellite alterations in serum or plasma DNA of patients with lung and head and neck cancers [81,82]. Subsequently, a prognostic role of MSI in plasma DNA was described in patients with small-cell lung cancer [83]. Considering the particular diagnostic, prognostic, and therapeutic significance of MSI status in many cancers, there is a growing need to develop novel approaches for MSI determination in liquid biopsies [128]. The highly fragmented nature of cfDNA and the small fraction of tumor DNA among total cfDNA in the body fluids, especially in the early stages of cancers [129], makes MSI determination in serum/plasma DNA technically challenging and requires highly sensitive methods. Standard-of-care classification of MSI/dMMR tumors is most frequently achieved using immunohistochemistry or PCR-based assays directed against a set of five microsatellite regions [128]. Mokarrom et al. compared real-time PCR with hybridization probes and high-performance liquid chromatography (HPLC) in assessing MSI status in tumors and sera of patients with colorectal cancer using two microsatellite loci (BAT-26 and BAT-25 markers), and MSI typing was shown to be a more accurate method for diagnosing MSI in CRC tumors, but not in serum cfDNA, compared with HPLC [84].

Recently, digital PCR and next-generation sequencing techniques were adapted for MSI testing. Silveira et al. evaluated the analytical performance of the previously described 3 markers (BAT-26, ACVR2A, and DEFB105A/B) in digital PCR with plasma samples from patients with advanced/metastatic colorectal and endometrial cancers [85], and determined the MSI with 100% sensitivity and specificity. In a recent study including patients with gastroesophageal adenocarcinoma, Boldrin et al. compared multiplex PCR, real-time PCR, and droplet digital PCR in MSI detection in formalin-fixed paraffin-embedded (FFPE) specimens and cfDNA and showed that only droplet digital PCR was able to detect MSI in cfDNA of patients with T3/T4 gastroesophageal adenocarcinoma [86], concluding that the droplet digital PCR assay could be considered as the most reliable and promising molecular approach to detect MSI in these patients.

Technical advances in NGS technology were recently employed in integrated cfDNA-based MSI detection, where a good overall concordance was observed between conventional MSI tissue-based testing and cfDNA-based MSI determination [130,131]. The concordance of cfDNA MSI with tissue PCR and next-generation sequencing was found to be significantly higher than in immunohistochemistry [131]. For patients with metastatic cancer treated with PD-1 blockade, MSI and high mutation burden in pretreatment plasma-predicted progression-free survival and longitudinal analysis of MSI sequencing led to the identification of patients with a durable response to PD-1 blockade. These analyses demonstrate the feasibility of cfDNA-based MSI detection as part of routine clinical practice to stratify patients with a better prognosis who are likely to benefit from targeted treatment [87].

### 3.6. Aberrant Telemore Sequences in Cell-Free DNA As a Biomarker of Cancer

Telomeres are tandem repeats of TTAGGG hexamers ranging from 10–15 kb in length at the ends of eukaryotic chromosomes. Telomeres have long been shown to prevent deleterious shortening of linear DNA of eukaryotic chromosomes during DNA replication and maintain chromosome integrity and genomic stability [132]. On the other hand, telomere shortening at chromosomal ends due to the restraints of the DNA replication process eventually leads to senescence or apoptosis, and acts as a tumor suppressor by restricting the replicative potential of primary cells. Tumor cells bypass this limitation primarily through the reactivation of telomerase, a hallmark of cancer, or via a recombination-based mechanism. However, even if experimental data on telomerase inhibition reveal that longer telomeres are more advantageous for cell survival, cancer cells often have paradoxically shorter telomeres compared with cells in the normal tissues [133]. Telomeric cfDNA levels or length analysis could be an informative genetic biomarker for many cancers. Wu and Tanaka [88] measured plasma telomeric cfDNA levels in patients with sporadic breast cancer and healthy individuals and found that plasma telomeric cfDNA levels decreased with age in healthy individuals, suggesting that cfDNA was likely derived from somatic cells in which telomere length shortens with increasing age. Intriguingly, telomeric cfDNA levels were significantly reduced in patients with breast cancer with no prior treatment compared with control individuals. The sensitivity and specificity for the telomeric cfDNA qPCR assay were 91.49% and 76.19%, respectively. Because tumor-suppressor gene products including BRCA1 and BRCA2 play an important role in telomere maintenance, Dey et al. hypothesized that plasma telomeric cfDNA levels could be associated with the mutation status of BRCA1 and 2 genes. They found that telomeric cfDNA levels were lower in unaffected BRCA1 and 2 mutation carriers than in age-matched controls [89], suggesting that plasma telomeric cfDNA levels were associated with breast cancer susceptibility.

Two further studies assessed cfDNA telomere length in gastric [90] and endometrial cancer [91]. In gastric cancer, with all measurements from baseline and different follow-up time-points, shortened telomeres were found to be significantly associated with gastric cancer risk (OR = 7.37, 95% CI: 2.06–26.32 for 1 unit shortening) [90]. Benati et al. evaluated the diagnostic performance of relative telomere length (i.e., telomere repeat copy number to single-gene copy number ratio) in cfDNA and found RTL to be significantly lower in endometrial patients with cancer [91]. In receiver operating characteristics (ROC) curve analysis, the diagnostic accuracy for endometrial cancer was 0.87 (95% CI: 0.79–0.95, *p* < 0.0001). These data on telomeric cfDNA are in accordance with the existence of paradoxically shorter telomeres in patients with cancer.

## 4. Concluding Remarks

Repeat DNA comprises more than 50% of the human genome and accomplishes many distinct functions. With respect to their genomic distribution, repeat DNA elements are divided into two mains groups: tandem repeats and interspersed repeats; the latter including retrotransposable elements. Advances in sequencing technologies enabled the definition of the composition of the repeat content of cfDNA more accurately. Retrotransposable elements and pericentric satellite DNA were found to be particularly overrepresented in the cfDNA population [35].

Applying repeat DNA in liquid biopsies provides many advantages. First, due to the high abundance of repeat element sequences in cfDNA, lower amounts of bodily fluids and template DNA are needed [51]. Second, their overrepresentation in cfDNA makes repeat DNA an ideal tool for liquid biopsy of patients with cancer and could be used to develop more accurate biomarkers with higher sensitivity and specificity. LINE-1 and ALU families, the main representatives of non-LTR interspersed retrotransposable elements, are the most used repeats in liquid biopsies of patients with cancer. The main areas of their employment include the quantitation of total cfDNA and the assessment of cfDNA integrity. In the analysis of global DNA methylation status, LINE-1 has been nearly the sole repeat element providing advantages over single-copy genes. Global hypomethylation of LINE-1 cfDNA was reported in many cancers and is associated with poor prognosis. Repeat DNA using LINE-1/ALU was also used in the detection of aneuploidy in cfDNA in patients with cancer, mainly with the amplicon sequencing approach [48].

Apart from LINE-1 and ALU, pericentric satellite DNA is a further repeat element type with significant potential as a biomarker in liquid biopsies of cancer because pericentric satellites have recently been shown to be the most overrepresented repeat (2230%) in cfDNA compared with their genomic abundance [35]. A further intriguing feature of these elements is that HSATII, a pericentric satellite repeat, was shown to expand in copy number in tumor cells via RNA-derived DNA intermediates [28], possibly leading to selective release into the circulation. Recently, we found significantly increased levels of HSATII, which was amplified by employing diluted plasma as a template without DNA extraction [80], because the lack of standardization of pre-analytical procedures such as DNA extraction impacts the outcomes of liquid biopsy measurements. The application of diluted plasma instead of extracted DNA as a template is a further advantage of applying repeat DNA in liquid biopsy and might help avoid variations and any bias introduced by DNA extraction. We believe that future studies with pericentric satellite DNA will enable the development of more reliable markers for noninvasive cancer screening and detection.

## Data Availability

Not applicable.
